# Long-Term Effects of a 35-Year Chronosequence of *Salix psammophila* Restoration on Soil Particle-Size Distribution and Erodibility in the Hobq Desert, Northern China

**DOI:** 10.3390/plants15152330

**Published:** 2026-07-29

**Authors:** Yifang Su, Haonian Li, Zhongju Meng, Zechen Shen, Xiaoyang Li

**Affiliations:** 1 College of Desert Control Science and Engineering, Inner Mongolia Agricultural University, Hohhot 010018, China; 2 State Key Laboratory of Water Engineering Ecology and Environment in Arid Area, Inner Mongolia Agricultural University, Hohhot 010018, China; 3 Desert Ecosystem Positioning Observation and Research Station in Hangjin, Ordos 017400, China; 4 School of Soil and Water Conservation, Beijing Forestry University, Beijing 100083, China

**Keywords:** *Salix psammophila*, soil particle size distribution, erodibility, multifractal dimensions, vegetation restoration process

## Abstract

In dryland ecosystems, the restoration of *Salix psammophila* shrubs plays a vital role in wind erosion control and sand stabilization. However, the temporal dynamics of soil particle-size distribution and erodibility during *S. psammophila* restoration remain poorly understood. To address this gap, we established a chronosequence of *S. psammophila* plantations in the Hobq Desert—a temperate desert in northern China—that had been restored for 6, 12, 15, 25, and 35 years, with adjacent shifting sand dunes serving as the control (CK). At each of the six sites, ten replicate plots were established, and soil samples were collected from the 0–20 cm layer, yielding a total of 60 samples. Multifractal parameters and the soil erodibility K factor were calculated to quantify the effects of stand age on particle-size distribution and erodibility. Principal component analysis (PCA) and a Random Forest model were then applied to factors associated for the observed changes. Compared with CK, soil nutrient and fine particle contents increased significantly with increasing shrub age, whereas pH and sand content declined continuously. Specifically, under *S. psammophila* plantations, organic carbon (OC), total nitrogen (TN), total phosphorus (TP), available phosphorus (AP), and alkali-hydrolysable nitrogen (AHN) contents increased continuously with stand age, while the soil texture became progressively finer. During long-term *S. psammophila* restoration, the ranges of the multifractal parameters *D*_0_, *D*_1_, *D*_2_ and *D*_1_/*D*_0_ were 0.82–0.91, 0.59–0.71, 0.50–0.58, and 0.70–0.78, respectively. *S. psammophila* restoration exhibited pronounced multifractal characteristics, which reduced the heterogeneity of the soil particle-size distribution and made the distribution more uniform, thereby resulting in a more stable soil structure and a more balanced ratio of fine to coarse particles. The soil erodibility K factor indicated that soil erosion resistance gradually increased with stand age, with a 23.71% reduction at 35 years compared with CK. Random Forest analysis identified organic carbon (OC), total nutrients (TN, TP), pH, soil particle-size fractions (clay, silt, sand), *D*_1_, *D*_2_, and vegetation characteristics (aboveground biomass, AGB; plant density, PD) as important predictor variables for soil erodibility (*p* = 0.01, R^2^ = 0.961). These findings provide new insights into the mechanisms by which long-term *S. psammophila* restoration improves soil structural stability and erosion resistance, offering a scientific basis for optimizing vegetation restoration and sustainable desert ecosystem management in arid regions.

## 1. Introduction

Desertification now covers 41% of the global terrestrial surface, constituting one of the most critical threats to terrestrial ecosystems. It not only destroys the structural integrity of soil habitats but also poses a potentially irreversible, long-term risk to regional and global ecological security [1,2]. In arid and semi-arid regions, vegetation restoration is widely acknowledged as the most effective bioengineering measure for rehabilitating degraded sandy lands and mitigating wind erosion and sand transport. As vegetation establishes, nutrients progressively accumulate beneath the canopy, generating a “fertile island effect” that fundamentally improves the physicochemical properties of aeolian sandy soils [3,4]. However, the primary mechanisms of soil improvement differ considerably among plant species. Shrubs, trees, and herbaceous plants exhibit pronounced differences in canopy interception, rooting depth, and associated traits, leading to distinct outcomes in soil nutrient status and particle-size distribution [5,6]. Therefore, elucidating the mechanisms by which specific pioneer constructive species in drylands modify sandy-soil microstructure is a prerequisite for designing species selection strategies and optimizing long-term ecosystem management in sandy regions [7,8].

*Salix psammophila* is a representative xerophytic shrub widely used in sand prevention and control systems in the arid and semi-arid regions of northern China. It is characterized by strong drought tolerance, outstanding resistance to wind erosion, and an extensive, complex root network, all of which enable it to play a vital role in drylands [9,10,11]. The establishment of large-scale *S. psammophila* plantations increases surface roughness and substantially attenuates near-surface erosive wind forces, while its well-developed fine roots continuously supply organic matter to the soil [12,13,14]. Previous studies have confirmed that vegetation restoration measures can effectively increase soil complexity and organic matter content, improve water-holding capacity, and significantly enhance nutrient accumulation [15,16]. However, existing studies have largely focused on short-term assessments of vegetation restoration effectiveness of shallow surface soils [17,18], whereas long-term successional studies spanning multiple decades remain scarce. Consequently, the long-term effects of *S. psammophila* plantations on soil properties are still unclear, and the soil rehabilitation benefits derived from their long-term sand fixation cannot yet be specifically quantified.

Particle-size distribution (PSD) is the most fundamental physical attribute of aeolian sandy soils, directly governing pore connectivity, wind erosion susceptibility, surface stability, and nutrient regulation capacity [19,20]. As vegetation restoration proceeds over time, vegetation interception and biochemical weathering jointly drive the progressive refinement of soil particles, manifested by a marked decline in the proportion of coarse sand and the concurrent accumulation of clay, silt, and very fine sand across soil horizons [4,21]. Li et al. [22] demonstrated that long-term windbreak and sand-fixing plantations in the desert-Yellow River transitional zone significantly increase silt and clay contents and promote a finer particle-size distribution. Conventional grain-size parameters can reflect the mechanical composition of soils, yet they fail to capture the complex scale-dependent distribution patterns and structural heterogeneity among different particle-size fractions. In recent years, fractal theory has been widely applied to characterize the spatial heterogeneity of soil structure [23,24]. Both single fractal dimensions and multifractal dimensions characterize the complexity of soil particle distribution using mathematical approaches.

Compared with conventional particle-size indices—such as the median grain size, sorting coefficient, and skewness—which primarily capture overall central tendency and dispersion, multifractal spectrum parameters, namely the capacity dimension (*D*_0_), information dimension (*D*_1_), and correlation dimension (*D*_2_), effectively characterize the breadth of the distribution, the degree of concentration, and the uniformity of soil particles across different size intervals [20,25]. By resolving local-scale properties within specific particle-size ranges, multifractal analysis describes the heterogeneous distribution of soil particles at multiple scales more accurately than traditional metrics. Previous studies have shown that vegetation establishment can alter the multifractal characteristics of soil particle-size distributions; for example, Li et al. [22] found that in arid regions, trees expanded the distribution range and increased heterogeneity and dispersion more markedly than shrubs, and they proposed multifractal parameters as complementary indicators for assessing changes in soil quality. To date, however, such studies have largely focused on comparing multifractal signatures among different plant species. During vegetation restoration in drylands, as stand age increases, concurrent improvements in habitat quality and windbreak and sand-fixation functions lead to non-uniform shifts in soil particle-size distribution over time. Introducing multifractal theory can therefore provide a more comprehensive understanding of the patterns of change in soil particle-size distribution throughout the restoration trajectory. At the same time, the heterogeneity in particle-size distribution induced by these evolving multifractal characteristics further modifies soil texture and structure, ultimately dictating the resistance of the soil to erosion. The soil erodibility K factor, a primary parameter for quantitatively assessing soil erosion resistance, is jointly influenced by factors such as particle-size composition and organic matter content [26,27]. Previous studies have indicated that an increase in fine particles supplies more erodible source material, leading to a distinct upward trend in the K factor and thus signifying reduced erosion resistance. However, systematic quantitative analysis of the dynamic responses of soil particle-size structural complexity and erodibility under long-term *S. psammophila* restoration remains lacking. Therefore, integrating the fractal characteristics of soil particle-size distribution with the soil erodibility K factor can help elucidate the formation mechanisms of soil erosion resistance at the microstructural scale [6,28]. Unlike previous studies, which focused primarily on short-term changes in soil physicochemical properties or conventional particle size metrics, this study integrates a long-term (35-year) vegetation restoration chronosequence with multifractal soil particle size distribution analysis and quantitative soil erosion assessment. This combined approach allows us to reveal not only temporal changes in soil texture during restoration but also the relationships between particle size heterogeneity, soil nutrient accumulation, vegetation development, and erosion resistance. As a result, this study provides new insights into the mechanisms by which long-term restoration of *S. psammophila* improves soil quality and ecosystem stability in desert environments.

We adopted a space-for-time substitution approach, using *S. psammophila* plantations of different ages to represent a restoration chronosequence. To minimize the inherent limitations of this approach, plot selection was restricted to sites with essentially uniform parent material and hydrothermal conditions. A 35-year chronosequence was established in the Hobq Desert, a temperate desert in northern China, consisting of shifting sand dunes (CK) and *S. psammophila* plantations aged 6, 12, 15, 25, and 35 years. At each site, surface soil samples (0–20 cm) were collected and vegetation restoration surveys were conducted simultaneously. Based on multifractal theory, we characterized the heterogeneity of soil particle-size distribution, and by combining measurements of soil nutrients and particle-size composition with the soil erodibility K factor, we assessed the dynamics of soil erodibility. The specific objectives of this study were to (1) reveal the temporal patterns of the multifractal parameters of soil particle-size distribution and the soil erodibility K factor, and (2) identify the dominant factors controlling soil particle-size distribution heterogeneity and the K factor. We hypothesized that increasing stand age of *S. psammophila* promotes the vegetation-mediated accumulation of fine soil particles and nutrients, thereby modifying the multifractal characteristics of soil particle-size distribution, enhancing soil structural stability, and increasing resistance to wind erosion.

## 2. Materials and Methods

### 2.1. Study Area Field Investigation

**Figure 1:**
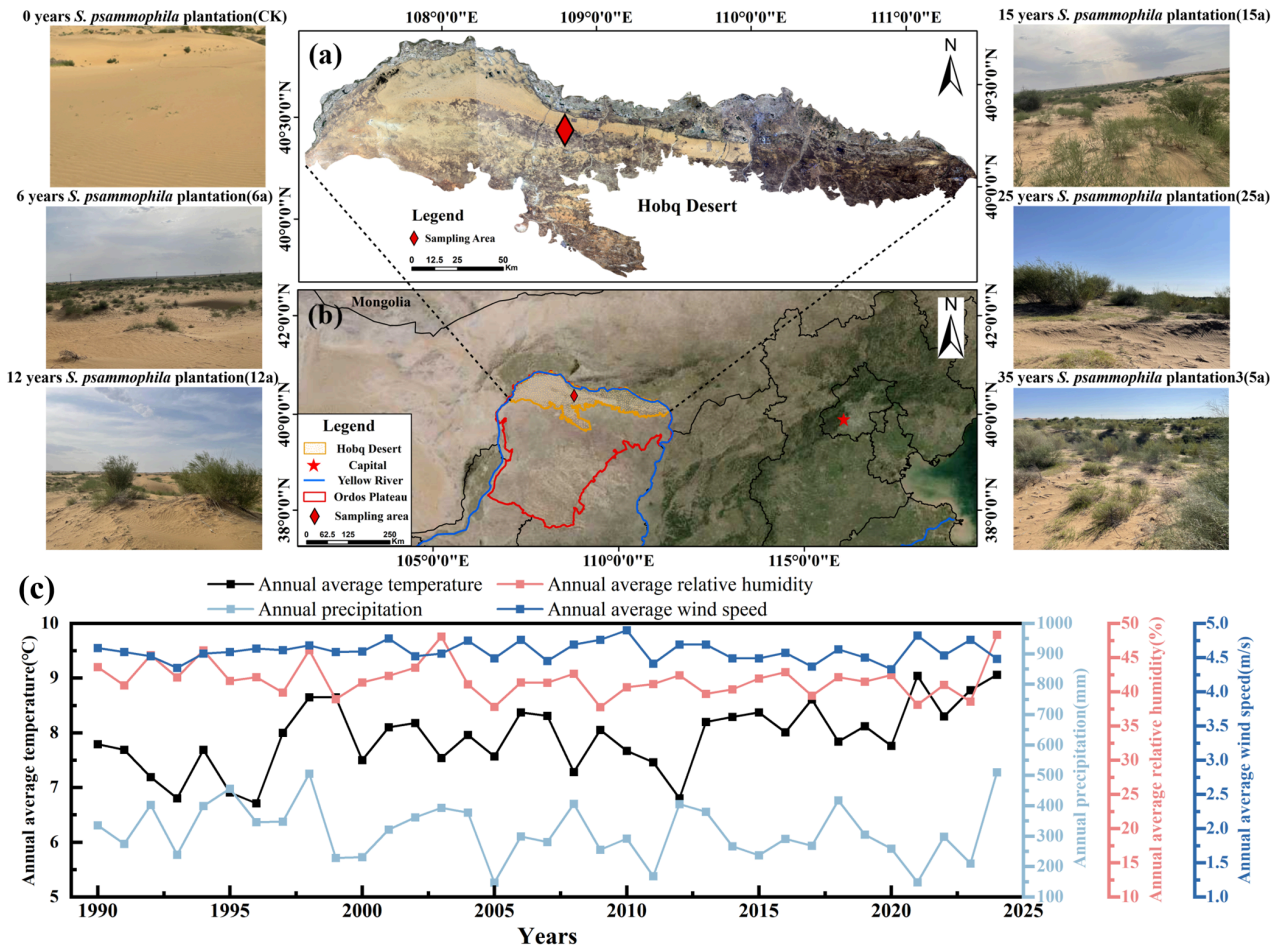
Location of the study area (**a**,**b**), a map of the *S. psammophila* restoration sites, and meteorological data for the study area (**c**).

The study area is located in the Hobq Desert, a temperate desert in northern China, and this research was conducted at the Hangjin Desert Ecological Positioning Observation Station of Inner Mongolia Agricultural University. The region has a mid-temperate arid monsoon climate. Over the past three decades, the mean annual precipitation averaged 316.00 mm, the mean annual temperature was 7.92 °C, and the mean annual wind speed was 4.59 m s^−1^; the corresponding meteorological data are shown in the Figure 1. Wind-sand activities are frequent in spring and autumn. Consequently, large-scale artificial windbreak and sand-fixing plantations have been established in the experimental area over the past three decades. These plantations are primarily composed of the shrub *S. psammophila*. Before the establishment of the mixed plantations, the original terrain mainly consisted of mobile dunes trending northwest-southeast, with heights ranging from 5 to 10 m. The dominant soil type in the study area is aeolian sandy soil.

### 2.2. Experimental Design and Soil Sample Collection

In this study, we employed a space-for-time substitution and established a chronosequence comprising a mobile dune control (CK) and five *S. psammophila* plantations aged 6, 12, 15, 25, and 35 years, representing successive restoration stages. To mitigate potential biases arising from spatial heterogeneity and the inherent limitations of the space-for-time approach, all selected plots were free of human disturbance, situated within the same landform, and located in close proximity. Vegetation characteristics—including plant height, cover, aboveground herbaceous biomass, *S. psammophila* density, and understory recovery—were recorded at each plot. As restoration progressed, herbaceous species such as *A. squarrosum*, *C. hyssopifolium*, *P. villosa*, *G. dasyphylla*, *A. desertorum*, and *S. alopecuroides* gradually colonized the understory (Table 1). With increasing stand age, the crown height of *S. psammophila* increased from 1.70 m at year 6 to 2.29 m at year 35. Vegetation cover rose from 12.99% to 55.99%, and aboveground herbaceous biomass increased from 13.39 to 40.21 g·m^−2^. *S. psammophila* density peaked at 0.05 individuals·m^−2^ in the 6-year plantation, then gradually declined and stabilized at 0.04 individuals·m^−2^ in older stands.

We established ten 20 m × 20 m plots in each of the five *S. psammophila* plantations (6, 12, 15, 25, and 35 years) and in the adjacent shifting sand area (CK), with all plots spatially separated to ensure independence. Within each plot, a five-point composite sampling strategy was adopted. Because wind erosion primarily acts on the surface layer, soil was collected from the 0–20 cm depth at each sampling point. The five subsamples from a given plot were thoroughly mixed to form one composite sample, yielding a total of 60 soil samples (6 treatments × 10 replicates). Additionally, ten undisturbed soil cores were randomly taken from each treatment using a cutting ring for the determination of soil bulk density and water content. All soil samples were transported to the laboratory, where visible plant roots and gravel were removed. The samples were air-dried, gently crushed, and passed through 2 mm and 0.15 mm sieves for subsequent analysis of soil particle-size composition, pH, organic carbon (OC), total nitrogen (TN), total phosphorus (TP), available phosphorus (AP), and alkali-hydrolysable nitrogen (AHN).

### 2.3. Soil Physicochemical Properties Determination

Soil bulk density and water content were determined using the core method, with samples oven-dried at 105 °C to constant weight and then weighed for calculation. Before the determination of soil particle size distribution, the samples were treated with 10% hydrogen peroxide (H_2_O_2_) to remove organic matter, and then ultrasonically dispersed with sodium hexametaphosphate solution, and then measured using a laser particle size analyzer (FRITSCH, Idar-Oberstein, Germany) over the range of 0–2000 μm and classified according to the USDA classification system as clay (<2 μm), silt (2–50 μm), and sand (50–2000 μm), with a variance of <0.5% [11]. Organic carbon (OC, g kg^−1^) was determined by the potassium dichromate external [22] heating method. Available phosphorus (AP, mg kg^−1^) was determined by the molybdenum-antimony colorimetric method after extraction with 0.5 mol L^−1^ NaHCO_3_ and measured using a UV spectrophotometer (ThermoFisher, MA, USA) [29]. The repeatability was ≤0.001 A, and the accuracy was ±0.004 A. Alkali-hydrolysable nitrogen (AHN, mg kg^−1^) was determined by the alkali diffusion method. Total nitrogen (TN, g kg^−1^) was measured by the Kjeldahl digestion method, and total phosphorus (TP, g kg^−1^) was determined by the molybdenum-antimony colorimetric method [30,31]. Soil pH was measured in a 5:1 water-soil mixture using a pH meter (ThermoFisher, Waltham, MA, USA) with an electrode to record the potential difference.

### 2.4. Calculation of Multifractal Parameters for Soil Particle Size Distribution

The generalized dimension D(q) and the singularity index α(q) of the multifractal spectrum were calculated according to the methods proposed by Rényi [32] and Chhabra et al. [33].

The generalized dimension D(q) is calculated as follows:

When q ≠ 1,

(1) $$D\left(q\right)=\underset{\varepsilon \to 0}{\lim}\frac{1}{q-1}\frac{\mathrm{lg}\left(\sum_{i=1}^{N\left(\varepsilon \right)}\mu_{i}{\left(\varepsilon \right)}^{q}\right)}{\log\varepsilon }$$

when q = 1,

(2) $$D_{1}=\underset{\varepsilon \to 0}{\lim}\frac{\sum_{i=1}^{N\left(\varepsilon \right)}\mu_{i}\left(\varepsilon \right)\mathrm{lg}\mu_{i}\left(\varepsilon \right)}{\log\varepsilon }$$

where represents the probability density weight index, an integer value ranging from –10 to +10. ε is the distribution interval of soil particle size, and μi(q,ε)is the q-order probability of the i-th interval.

The singularity index α(q) of the multifractal spectrum is calculated as follows:

(3) $$u_{i}\left(q,\varepsilon \right)=\frac{{u_{i}\left(\varepsilon \right)}^{q}}{\sum_{i=1}^{N\left(\varepsilon \right)}\mu_{i}{\left(\varepsilon \right)}^{q}}$$

(4) $$\alpha \left(q\right)=\underset{\varepsilon \to 0}{\lim}\frac{\sum_{i=1}^{N\left(\varepsilon \right)}u_{i}\left(q,\varepsilon \right)lg\mu_{i}\left(\varepsilon \right)}{lg\varepsilon }$$

The multifractal spectrum f(α) is defined as follows:

(5) $$f\left(\alpha \left(q\right)\right)=\underset{\varepsilon \to 0}{\lim}\frac{\sum_{i=1}^{N\left(\varepsilon \right)}u_{i}\left(q,\varepsilon \right)lgu_{i}\left(q,\varepsilon \right)}{lg\varepsilon }$$

When q = 0, 1, and 2, the calculated generalized dimension spectrum D(q) corresponds to *D*_0_ (capacity dimension), *D*_1_ (information dimension), and *D*_2_ (correlation dimension), respectively. Among the multifractal parameters, *D*_0_ reflects the richness and range of the soil particle size distribution; a higher *D*_0_ value indicates a broader range of soil particle sizes; *D*_1_ reflects the uniformity of the particle size distribution; a higher *D*_1_ value indicates a more dispersed distribution; *D*_2_ reflects the degree of aggregation and structural stability of the major particle fractions; a higher *D*_2_ value indicates a more uniform distribution. The *D*_1_/*D*_0_ ratio indicates whether there is an abnormal enrichment of extreme particle sizes in localized areas. The width of the multifractal singularity spectrum, Δα = (αmax − αmin), quantifies the heterogeneity of soil particle-size distribution within the fractal structure. A larger Δα signifies greater heterogeneity of the soil particle-size distribution. Δƒ[(ƒ(αmax) − ƒ(αmin)] reflects the shape of the multifractal spectrum. When Δƒ < 0, small-diameter particles play a dominant role in determining the overall soil particle distribution; conversely, Δƒ > 0 indicates the opposite scenario. It should be noted that the generalized fractal dimensions *D*_0_, *D*_1_, *D*_2_, and *D*_1_/*D*_0_ do not characterize exactly the same features of particle-size distribution as the multifractal singularity spectrum parameters Δα and Δƒ. *D*_0_, *D*_1_, and *D*_2_ primarily reflect the overall coverage range, information uniformity, and correlative evenness of the soil particle-size distribution, whereas Δα and Δƒ focus more on local differences among particle-size fractions and spectral asymmetry.

### 2.5. EPIC Soil Erodibility Model

In this study, the EPIC (Erosion-Productivity Impact Calculator) model was selected to assess soil erodibility in desert areas [34]. The calculation formula is as follows:

(6) $$K=\left\{0.2+0.3exp\left[-0.0256Sa\left(1-\frac{Si}{100}\right)\right]\right\}\times {\left(\frac{Si}{Ci+Si}\right)}^{0.3}\times \left(1.0-\frac{0.25C}{C+exp\left(3.72-2.95C\right)}\right)\;\times \left(1.0-\frac{0.75Sn}{Sn+exp\left(-5.51+22.9Sn\right)}\right)$$

where Sa is the sand, Si is the silt, Ci is the clay, Sn is the percentage of silt and clay, and C is the organic carbon. This model offers significant advantages in terms of parameter fit and ecological impact assessment, and is particularly well-suited for modeling complex erosion processes in arid and semiarid ecosystems [35].

### 2.6. Data Analysis

One-way analysis of variance (ANOVA) was performed using SPSS 26.0 (IBM Corp., Armonk, NY, USA), followed by Duncan’s multiple range test for post hoc multiple comparisons at a significance level of *p* < 0.05, to identify significant differences in the selected indicators among *S. psammophila* plantations of different stand ages. To reveal the intrinsic relationships among the multifractal parameters of soil particle-size distribution, the soil erodibility K factor, and environmental factors, Spearman’s rank correlation analysis was conducted. The sign of the correlation coefficient (r) indicates the direction of the relationship; *p* < 0.05 was considered statistically significant, and *p* < 0.01 was considered highly significant. In addition, principal component analysis (PCA) and Random Forest modeling were performed on all variables. PCA was conducted using Origin 2024 (OriginLab Corporation, Northampton, MA, USA), and the Random Forest model was implemented using the Random Forest package in R version 4.5.3, with the number of decision trees (ntree) set to 1000 to ensure error stability, followed by ranking the importance of the driving factors.

## 3. Results

### 3.1. Distribution Characteristics of Soil Physicochemical Properties Under Different Stand Age of Checkerboard Barriers of S. psammophila

#### 3.1.1. Dynamics of Soil Physical Properties During the Restoration of *S. psammophila*

The improvement of soil physical properties directly reflects the restoration of *S. psammophila*. As shown in Table 2, in the control mobile dunes (CK), soil water content was only 0.03%, bulk density was as high as 1.56 g cm^−3^, and the soil structure was loose. At the early stage of restoration (6a), soil water content significantly decreased to 0.03% (*p* < 0.05). Soil bulk density exhibited a sustained and significant decreasing trend, gradually declining from 1.56 g cm^−3^ in CK to 1.49 g cm^−3^ at 35a (*p* < 0.05), a decrease of 4.40%, indicating that soil pore conditions progressively improved toward a loose, porous, and highly permeable structure.

The evolution of soil particle-size composition was particularly pronounced. As shown in Table 2, sand content in CK was as high as 98.74%, while clay and silt contents were only 0.30% and 0.94%, respectively. In the 6-year *S. psammophila* plantation, clay and silt contents were 0.09% and 0.67%, respectively, and sand content was 99.23%. However, starting from 12 years (12a), the soil gradually became finer: clay and silt contents increased to 0.30% and 1.43%, respectively, and sand content decreased to 98.23%. Thereafter, the clay and silt contents increased exponentially, reaching 1.43% and 6.89% at 35a, which were 4.8 and 7.4 times those of CK, respectively; meanwhile, sand content dropped sharply to 91.67%, a decrease of 7.20% compared with CK.

#### 3.1.2. Analysis of Soil Nutrient Distribution Characteristics Under *S. psammophila* Plantations of Different Stand Ages

**Figure 2:**
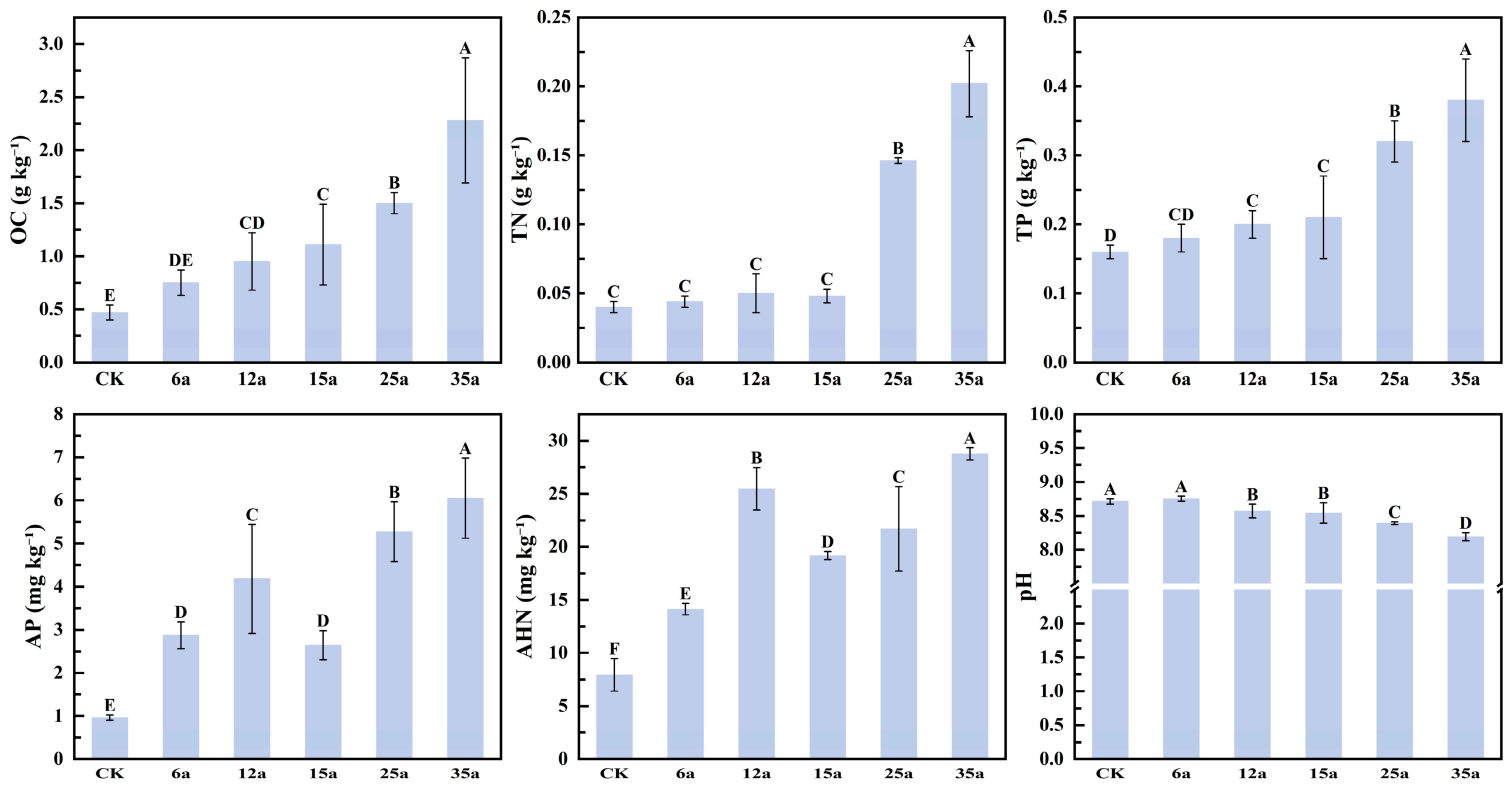
Distribution of soil nutrients under *S. psammophila* plantations of different stand ages. Note: Data are presented as mean ± SD (*n* = 10). Different uppercase letters within each column indicate significant differences among stand ages according to Duncan’s multiple range test (*p* < 0.05). Abbreviations: Organic carbon (OC), total nitrogen (TN), total phosphorus (TP), available phosphorus (AP), alkali-hydrolysable nitrogen (AHN), pH.

As shown in Figure 2, OC content in CK was extremely low, averaging approximately 0.47 g kg^−1^, indicative of an extremely infertile state. At 6 years, OC increased slightly to about 0.75 g kg^−1^, but the increase was not significant. The true rapid accumulation phase began after 12 years, at which point OC rose to 0.95 g kg^−1^, reaching 1.11 g kg^−1^ at 15 years. At 25 and 35 years, OC content surged to 1.51 g kg^−1^ and 2.28 g kg^−1^, respectively (*p* < 0.05), representing 3.2 times and 4.9 times increases relative to CK, indicating that litter return and microbial carbon sequestration effects strengthened markedly over time with vegetation restoration. TN displayed a similar trend, reaching 0.20 g kg^−1^ at 35 years, which was 5.0 times that of CK. TP and AP contents also peaked at 35 years. Notably, AHN showed the most remarkable increase, soaring from approximately 7.92 mg kg^−1^ in CK to 28.76 mg kg^−1^ at 35 years (*p* < 0.05), a 3.6 times increase. Soil pH exhibited an opposite declining trend. The pH in CK was about 8.71, indicating strong alkalinity. As restoration progressed, pH decreased gradually, and by 35 years, soil pH had declined from 8.71 in CK to 8.19 (*p* < 0.05), demonstrating that long-term vegetation restoration significantly reduced soil alkalinity.

### 3.2. Variation Patterns of Fractal Dimensions of Soil Particle-Size Distribution During S. psammophila Restoration

#### 3.2.1. Single Fractal Dimension

As shown in Table 3, the D value of CK was only 2.06, indicating a relatively simple soil particle-size distribution with coarse particles. At 6 years, the D value dropped sharply to 1.89 (*p* < 0.05), the lowest among all treatments, suggesting that fine particles were lost due to wind sorting at the early restoration stage, resulting in a more uniform particle-size distribution. Thereafter, with the progressive accumulation of clay and silt particles, the D value began to increase continuously: it was 2.06 and 2.07 at 12 and 15 years, respectively, recovering to a level close to that of CK. It rose significantly to 2.14 at 25 years, and reached the maximum value of 2.35 at 35 years (*p* < 0.05), representing a 14.1% increase compared with CK.

#### 3.2.2. Multifractal Dimensions

**Figure 3:**
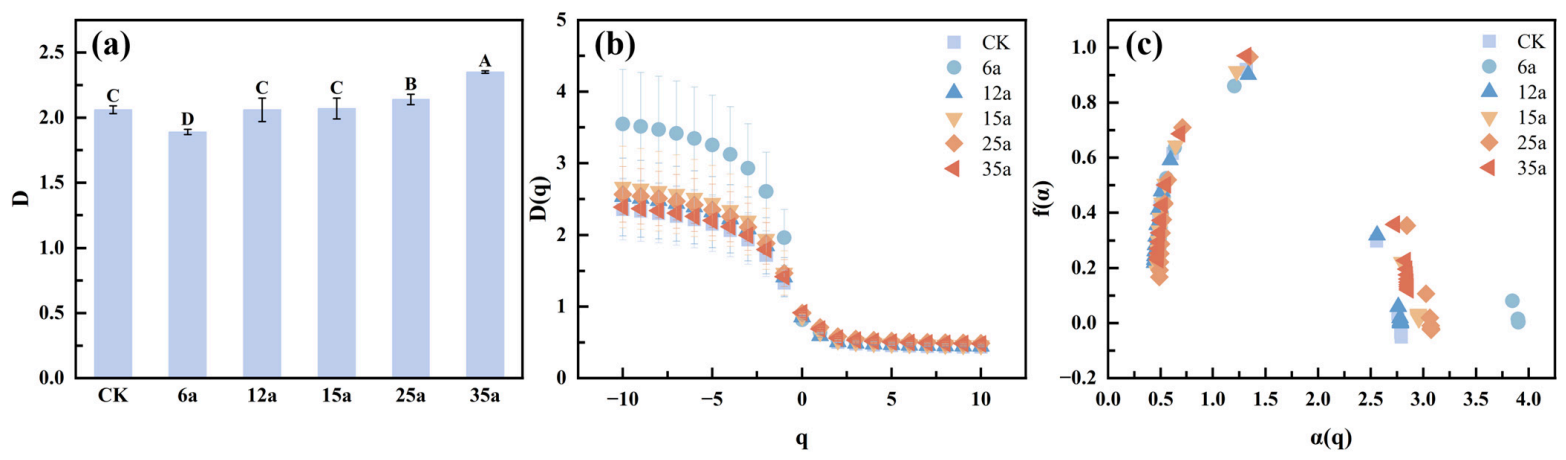
Single fractal dimension (**a**), generalized dimension spectrum (**b**), and singularity spectrum (**c**) of soil particle-size distribution under different stand ages. Note: In panel (**b**), the x-axis is the moment order *q* and the y-axis is the generalized dimension D(q). In panel (**c**), the x-axis represents the singularity exponent α(q), which characterizes the local scaling properties of particle distribution, and the y-axis represents the multifractal spectrum *f*(α), indicating the fractal dimension of the subset with exponent α. Data are presented as mean ± SD (*n* = 10). Different uppercase letters within each column indicate significant differences among stand ages according to Duncan’s multiple range test (*p* < 0.05).

As shown in Figure 3, the soil particle-size distribution under *S. psammophila* plantations of different stand ages exhibited differences in the complexity and uniformity of the distribution. The generalized dimension spectra D(q) of all treatments displayed a typical inverse S-shaped decreasing curve. The variation amplitude in the negative domain (q= −10 to 0) was greater than that in the positive domain (q= 0 to 10). The D(q) curve of the 35-year treatment in the q > 0 region shifted markedly upward and tended to flatten. As shown in Table 3, we systematically quantified the multifractal characteristics of soil particle-size distribution by calculating the capacity dimension (*D*_0_), information dimension (*D*_1_), and correlation dimension (*D*_2_), together with the *D*_1_/*D*_0_ ratio. *D*_0_ generally exhibited a decreasing-then-increasing trend. *D*_0_ of CK was 0.87, and it decreased significantly to 0.82 and 0.85 at 6 and 12 years, respectively (*p* < 0.05), indicating a narrowing of the particle-size distribution range. However, with increasing stand age, *D*_0_ showed a continuous increase after 15 years, reaching 0.91 at 35 years, respectively, which were significantly higher than that of CK (*p* < 0.05). The *D*_1_ and *D*_1_/*D*_0_ of CK were 0.62 and 0.71, respectively. With *S. psammophila* restoration, *D*_1_ at 6, 12, 25, and 35 years was significantly higher than that of CK. Furthermore, the *D*_1_/*D*_0_ ratios of all treatments were less than 1, which theoretically confirmed that the soil particle-size distribution at each restoration stage exhibited significant multifractal characteristics. The *D*_2_ of CK was 0.50. Except at 12 years, *D*_2_ values under *S. psammophila* plantations of all ages were significantly higher than that of CK (*p* < 0.05), implying that *S. psammophila* restoration made the soil particle-size distribution more uniform compared to mobile.

We constructed the singularity spectrum of soil particle sizes based on α(q) and ƒ(α). As shown in Figure 3c, all spectra exhibited an asymmetric parabolic shape. Differences in α(q) and ƒ(α) were observed among different *S. psammophila* stand ages, and Δƒ > 0, indicating that the coarse fraction dominated the soil particle-size distribution pattern. In addition, Δα differed among stand ages, reflecting differences in the uniformity of particle-size distribution, with Δα at 6 years being significantly larger than that at other ages. Compared with the generalized fractal dimensions, these variations in the multifractal singularity spectrum parameter Δα revealed the differentiation within different particle-size intervals in the soil, particularly the pronounced heterogeneity between fractions with high and low contents.

### 3.3. Variation Patterns of Soil Erodibility K Factor During S. psammophila Restoration

**Figure 4:**
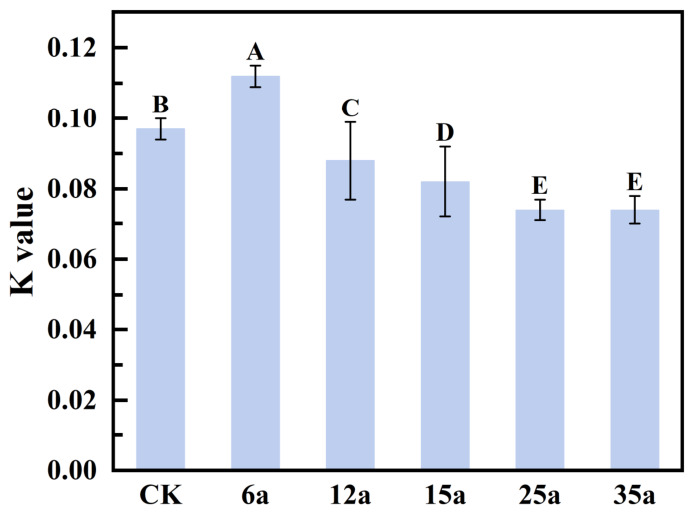
Comparison of soil erodibility K factor under *S. psammophila* plantations of different stand ages. Note: Data are presented as mean ± SD (*n* = 10). Different uppercase letters within each column indicate significant differences among stand ages according to Duncan’s multiple range test (*p* < 0.05).

The soil erodibility K factor calculated based on the EPIC model comprehensively reflects changes in soil erosion resistance (Figure 4). The mean K factor of CK was 0.10, indicating that mobile dunes are highly susceptible to erosion. At 6 years, the K factor was 0.11, significantly higher than that of CK (*p* < 0.05). From 12 years onward, the K factor entered a sustained and significant decline, with values of 0.09, 0.08, 0.07, and 0.07 at 12, 15, 25, and 35 years, respectively (*p* < 0.05), representing decreases of 9.28%, 15.46%, 23.71%, and 23.71% relative to CK. This change was strongly coupled with soil fining and nutrient enrichment. The effects of different *S. psammophila* stand ages on soil erodibility or structural stability differed markedly; overall, vegetation restoration reduced soil erodibility.

### 3.4. Effects of Environmental Factors on Fractal Dimensions of Soil Particle-Size Distribution and Soil Erodibility K Factor

**Figure 5:**
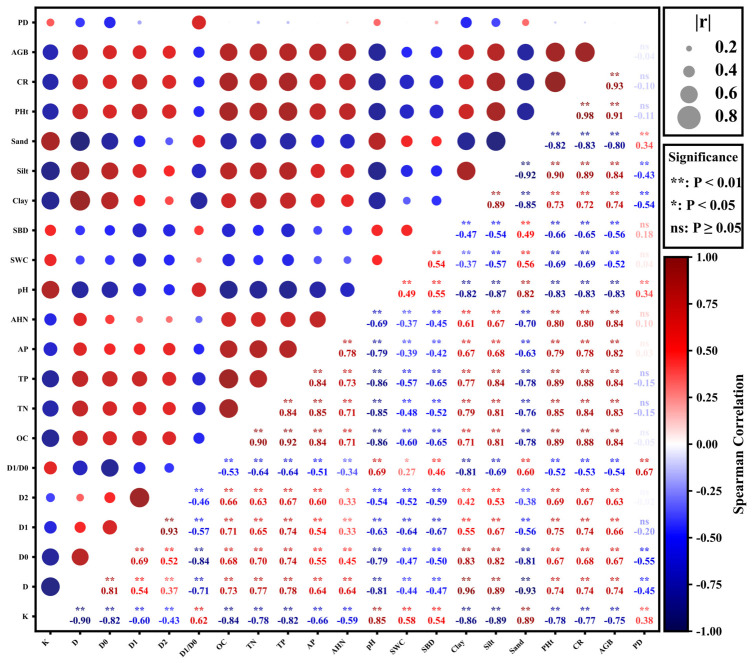
Correlation analysis among multifractal dimensions of soil particle-size distribution, soil erodibility K factor, and environmental factors (red indicates positive correlation, blue indicates negative correlation). Significance levels: * *p* < 0.05, ** *p* < 0.01. Abbreviations: capacity dimension (*D*_0_), information dimension (*D*_1_), correlation dimension (*D*_2_), organic carbon (OC), total nitrogen (TN), total phosphorus (TP), available phosphorus (AP), alkali-hydrolysable nitrogen (AHN), pH, soil water content (SWC), soil bulk density (SBD), plant height (PHt), vegetation coverage (CR), aboveground herbaceous biomass (AGB), plant density (PD).

In this study, Spearman correlation analysis was performed on soil physicochemical properties, vegetation characteristics, multifractal dimensions, and the soil erodibility K factor for the CK, 6a, 12a, 15a, 25a, and 35a plots. The correlation matrix (Figure 5) showed that the fractal dimensions of soil particle-size distribution (including D, *D*_0_, *D*_1_, and *D*_2_) exhibited significant positive correlations (*p* < 0.05) with soil nutrients (including OC, TN, TP, AP, and AHN), fine soil particles (i.e., clay and silt), and vegetation growth characteristics (including PHt, CR, and AGB), with correlation coefficients ranging from 0.25 to 0.91. The fractal dimensions of soil particle-size distribution (including D, *D*_0_, *D*_1_, and *D*_2_) showed significant negative correlations (*p* < 0.05) with pH, SWC, SBD, and sand content, with correlation coefficients ranging from –0.78 to –0.19. In addition, *D*_0_ displayed a significant negative correlation with PD (*p* < 0.05). *D*_1_/*D*_0_ showed significant negative correlations (*p* < 0.05) with soil nutrients (including OC, TN, TP, AP, and AHN), fine soil particles (i.e., clay and silt), and vegetation growth characteristics (including PHt, CR, and AGB), with correlation coefficients ranging from –0.88 to –0.23. *D*_1_/*D*_0_ exhibited significant positive correlations (*p* < 0.05) with pH, SWC, SBD, sand content, and PD, with correlation coefficients ranging from 0.21 to 0.75. The soil erodibility K factor showed significant negative correlations (*p* < 0.05) with soil multifractal parameters, soil nutrients, fine soil particles, and vegetation growth characteristics (including PHt, CR, and AGB).

Principal component analysis (PCA) further revealed that PC1 and PC2 explained 68.4% and 12.7% of the total variance, respectively (cumulatively 81.1%), indicating that the first two principal components adequately captured the integrated variation in vegetation growth characteristics, soil particle-size composition, physicochemical properties, multifractal parameters, and the K factor across different stand ages of *S. psammophila*. The directional distribution of the indicators represented their interrelationships, which was consistent with those shown in the figure. Regarding sample distribution, different stand ages exhibited a certain degree of differentiation in PCA space, though overlaps among samples persisted, suggesting that treatment effects might exist but with limited separation. Overall, the increases in fine soil fractions and nutrient contents, as well as *S. psammophila* growth, were consistent with the increases in multifractal dimensions.

**Figure 6:**
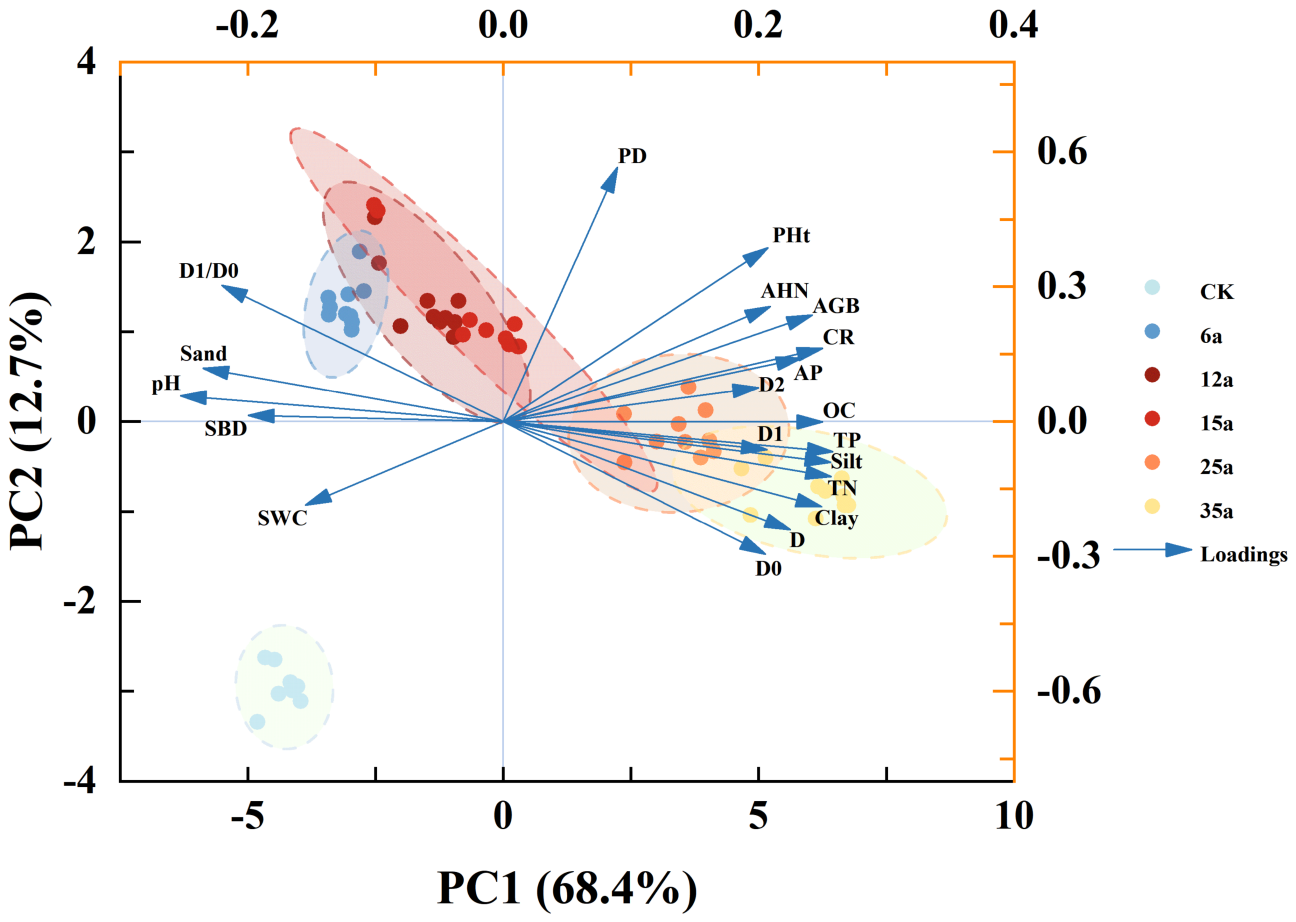
Principal component analysis (PCA) showing the relationships among soil particle-size multifractal parameters, soil erodibility (K factor), and environmental variables. Arrows represent the loadings of each variable on the first two principal components, indicating the direction and strength of their contribution to the ordination. Abbreviations: organic carbon (OC), total nitrogen (TN), total phosphorus (TP), available phosphorus (AP), alkali-hydrolysable nitrogen (AHN), pH, soil water content (SWC), soil bulk density (SBD), capacity dimension (*D*_0_), information dimension (*D*_1_), correlation dimension (*D*_2_), plant height (PHt), vegetation coverage (CR), aboveground herbaceous biomass (AGB), plant density (PD).

As shown in Figure 6, principal component analysis (PCA) was performed on the multifractal dimensions of soil particle-size distribution, the soil erodibility K factor, and environmental factors under CK, 6a, 12a, 15a, 25a, and 35a conditions. The results showed that PC1 and PC2 explained 68.4% and 12.7% of the total variance, respectively, cumulatively accounting for 81.1%. The correlations among indicators (Figure 6) were consistent with the Pearson correlation analysis (Figure 5), and the angles between indicator vectors reflected the magnitude of correlation coefficients. The K factor, sand content, and pH were significantly negatively correlated with soil nutrients, vegetation characteristics, and soil multifractal parameters.

**Figure 7:**
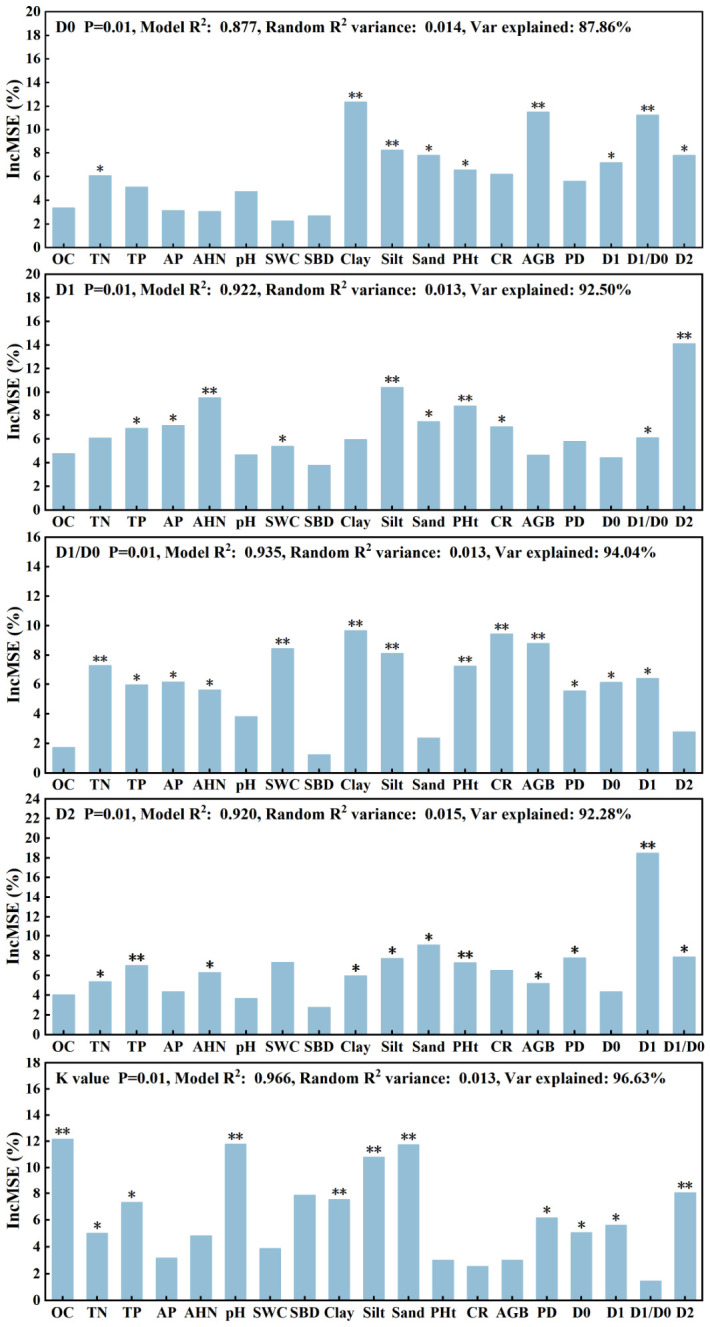
Variable importance derived from Random Forest models for soil multifractal parameters and the soil erodibility (K) factor. Significance levels: * *p* < 0.05, ** *p* < 0.01. Abbreviations: organic carbon (OC), total nitrogen (TN), total phosphorus (TP), available phosphorus (AP), alkali-hydrolysable nitrogen (AHN), pH, soil water content (SWC), soil bulk density (SBD), plant height (PHt), vegetation coverage (CR), aboveground herbaceous biomass (AGB), plant density (PD), capacity dimension (*D*_0_), information dimension (*D*_1_), correlation dimension (*D*_2_).

The Random Forest models for each multifractal parameter and for the K factor were all statistically significant (all model *p* < 0.05), with R^2^ values ranging from 0.90 to 0.96, explaining 90.3–96.4% of the variance, and residual variance as low as 0.01–0.02, indicating both high explanatory power and robust stability (Figure 7). For the capacity dimension (*D*_0_), clay content was the most important variable and reached a highly significant level (*p* < 0.01), while total nitrogen (TN), silt, sand, and pH also showed significant associations (*p* < 0.05), and aboveground biomass (AGB) was significantly correlated (*p* < 0.05). The information dimension (*D*_1_) was highly significantly related to silt, total phosphorus (TP), and pH (*p* < 0.01), and significantly related to available phosphorus (AP), clay, and plant density (PD) (*p* < 0.05). For the *D*_1_/*D*_0_ ratio, vegetation cover (CR) had the highest increase in mean squared error (IncMSE); clay, silt, and AGB reached highly significant levels (*p* < 0.01), while TN, TP, alkali-hydrolysable nitrogen (AHN), soil water content (SWC), and PD were significant (*p* < 0.05). The correlation dimension (*D*_2_) was significantly correlated with TN, TP, clay, silt, pH, CR, and PD (*p* < 0.05). Collectively, these results indicate that total nutrients, fine particle fractions, and plant growth characteristics are closely linked to the multifractal signature of soil particle-size distribution. With respect to the soil erodibility K factor, silt content emerged as the most important predictor; organic carbon (OC), TN, TP, pH, soil bulk density (SBD), clay, and sand were also significantly correlated (*p* < 0.05). Furthermore, the K factor showed highly significant correlations with the multifractal parameters *D*_1_, *D*_2_, and PD (*p* < 0.01). Overall, as *S. psammophila* stands develop in this sandy landscape, the soil erodibility K factor becomes tightly coupled to total soil nutrients, pH, soil texture and structure, and vegetation density.

## 4. Discussion

### 4.1. Response of Soil Physicochemical Properties to Long-Term S. psammophila Restoration

Our results demonstrated that long-term restoration of *S. psammophila* plantations in the Hobq Desert substantially altered soil physicochemical properties, with the degree of improvement showing a strong dependence on stand age. Soil organic carbon (OC), total nitrogen (TN), total phosphorus (TP), available phosphorus (AP), and alkali-hydrolysable nitrogen (AHN) all increased consistently with increasing stand age (Figure 2), a pattern that agrees well with previous reports of shrub-mediated soil improvement in drylands [3,4,5]. Notably, the most pronounced nutrient accumulation occurred after 12 years of restoration; by year 35, OC content reached 2.67 g/kg, representing a 4.6-fold increase relative to the control (CK). This gradual nutrient enrichment is not simply a direct consequence of vegetation growth but rather reflects the combined effects of multiple interacting mechanisms, including litter accumulation, suppressed surface wind erosion, and progressive microclimate amelioration [36].

Of particular note is that at the 6-year site, clay and silt fractions were unexpectedly lower than those in the control (CK), while the sand fraction reached as high as 99.225%. We attribute this to the limited capacity of the sparse, short-statured vegetation to suppress wind erosion at this early stage. Previous work has established that vegetation improves soil particle-size composition through root activity and litter input [22] and increases surface roughness to achieve windbreak and sand-fixation effects [29]. However, during the initial phase of establishment, fine particles can still be selectively removed by wind from around the plants. Using computational fluid dynamics (CFD), Gonzales [37] demonstrated that vegetation canopies retain particles in a size-selective manner; in the early stages of shrub restoration, the canopy preferentially traps coarser particles, which can leave surface soils locally enriched in sand and depleted in fines.

In the later stages of restoration, as the *S. psammophila* canopy matured, the accumulation of fine particles accelerated markedly; by year 35, clay and silt contents reached 4.8 and 7.4 times the values in CK, respectively. This long-term refinement of soil texture resulted not only from direct plant-mediated sand fixation but also from the replenishment of fine particles by sustained atmospheric dust deposition and eolian processes [38]. The substantial increase in alkali-hydrolysable nitrogen at 35 years (from 8.19 mg kg^−1^ in CK to 28.78 mg kg^−1^) was driven by the distinctive microclimate generated by the mature shrub canopy and its extensive root network. This microclimate buffered surface temperature fluctuations and helped conserve soil moisture, providing favorable microhabitats for the colonization and survival of understory species. The subsequent establishment of legumes such as *Sophora alopecuroides* in the understory community (Table 1) further enhanced nitrogen accumulation through biological nitrogen fixation [39,40].

Soil pH declined steadily from 8.71 in the control (CK) to 8.19 in the 35-year plantation, a trend likely driven by the accumulation of organic acids from litter decomposition and enhanced microbial metabolism. Soil bulk density concurrently decreased from 1.56 g cm^−3^ in CK to 1.49 g cm^−3^ at 35 years (Table 2), indicating a progressive increase in soil porosity. Although soil nutrients and structure showed a clear inflection point after approximately 25 years, long-term monitoring remains necessary to determine whether soil properties had reached a stable equilibrium by year 35.

### 4.2. Evolution of Single and Multifractal Parameters of Soil Particle-Size Distribution During S. Psammophila Restoration

The application of fractal theory provides a more nuanced perspective for understanding the structural reorganization of soil particles during long-term vegetation restoration. The single fractal dimension (D) dropped sharply from 2.06 in CK to 1.89 at 6 years, the lowest value among all treatments (Table 3). This initial decline reflects the preferential loss of fine particles through wind sorting, leading to a more uniform and simplified particle composition [10,41]. This observation is consistent with the decline in *D*_0_ at 6 years, which indicates a narrowing of the particle-size distribution range. Thereafter, the D value increased steadily with stand age, reaching 2.35 at 35 years, a trend that closely paralleled the accumulation of clay and silt fractions and signaled a progressive increase in the complexity of soil particle composition. This upward shift in the single-scale fractal dimension is consistent with patterns documented for other sand stabilization measures in arid and semi-arid regions—such as grassland restoration and straw checkerboard barriers—affirming that vegetation restoration generally enhances the structural complexity of soil systems [29,42].

Multifractal parameters offer a more detailed, micro-scale perspective on the dynamics of soil particle-size distribution (PSD). The initial decline and subsequent rise in the capacity dimension (*D*_0_) mirror the two-stage pattern exhibited by the single-scale fractal dimension (D). By year 35, both the information dimension (*D*_1_) and the correlation dimension (*D*_2_) were significantly higher than those in CK. These shifts are not merely numerical; they indicate that long-term restoration enhanced the dispersion and uniformity of the particle-size distribution, thereby reducing the overwhelming dominance of coarse particles. The generalized dimension spectra, D(q), of all treatment groups displayed a characteristic inverse-S shape, and their asymmetry confirms that fine particles contribute more strongly to the system’s structure than coarse particles. The broadening of the singularity spectrum (Δα increased from 1.53 to 2.45) directly reflects the restoration-driven intensification of microhabitat heterogeneity and the associated increase in soil structural complexity [43,44].

**Figure 8:**
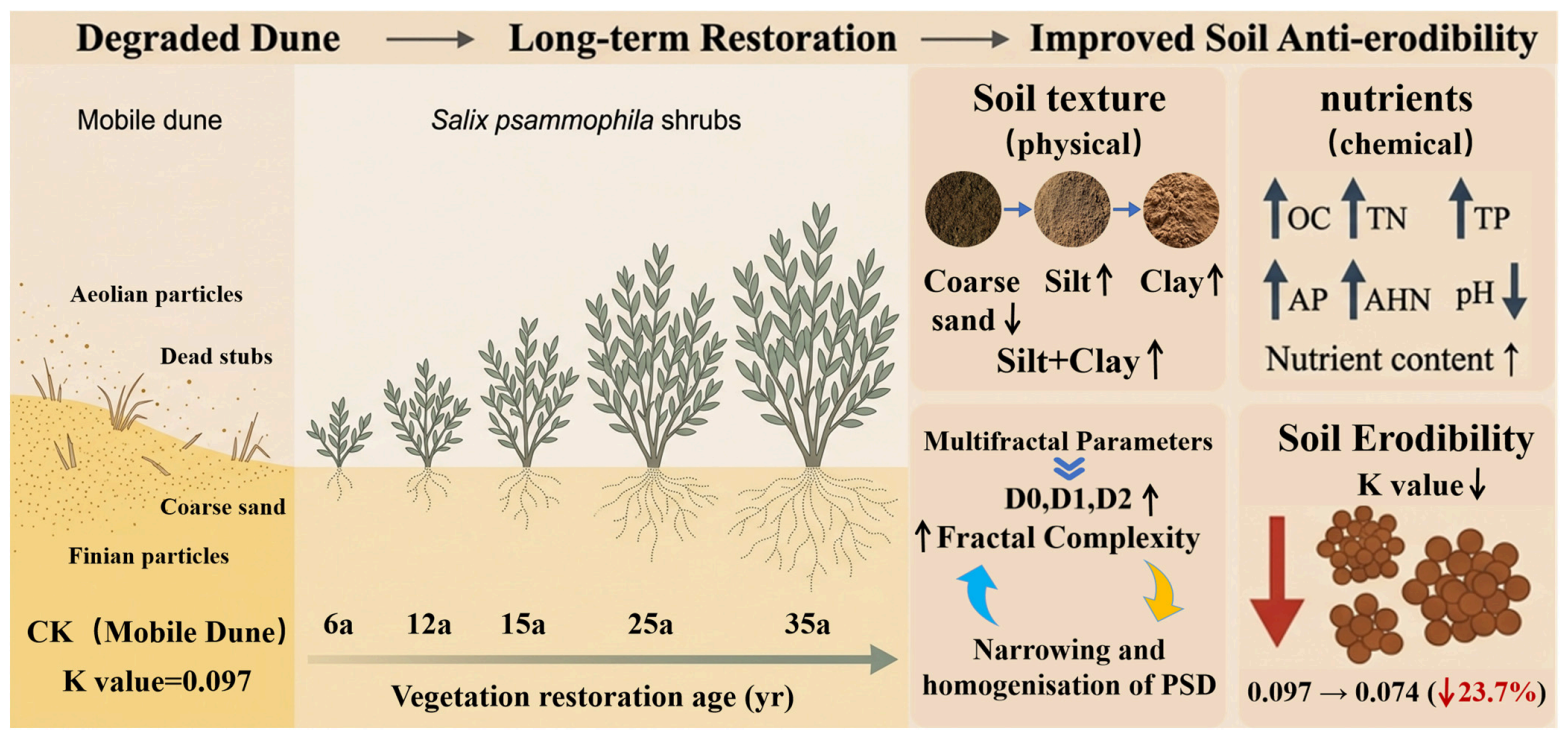
Conceptual diagram illustrating the effects of *S. psammophila* restoration on soil particle-size distribution and erodibility.

Notably, our Random Forest analysis identified aboveground biomass (AGB), plant density (PD), and vegetation cover (CR) as the primary vegetation traits governing the fractal evolution described above. Higher AGB promotes litter accumulation and root exudation, thereby enhancing the aggregation of fine particles and driving the progressive increases in D and *D*_0_ over time [45,46]. Concurrently, the proliferation of herbaceous vegetation and greater cover increase surface aerodynamic roughness, reduce near-surface wind shear velocity, and mitigate the selective removal of fine particles, thereby fostering a more homogeneous particle-size distribution—a shift reflected in the broadening of Δα [46,47]. In contrast, the influence of plant density on multifractal parameters operates in an opposing direction. As *S. psammophila* stands mature, the limited water-carrying capacity of sandy habitats constrains the recruitment of additional individuals within a finite area [48]. During early recovery stages, although higher plant density effectively promotes the deposition of coarser particles, wind erosion of fine particulates persists in the gaps between shrubs [37]. Collectively, these vegetation-mediated mechanisms—spanning from biophysical interception to biochemical aggregation—account for the gradual increase in multifractal complexity and reveal a direct causal pathway linking plant community traits to soil structural recovery during *S. psammophila* restoration (Figure 8).

### 4.3. Driving Mechanisms of Enhanced Soil Erosion Resistance

The soil erodibility K factor, calculated using the EPIC model, declined markedly with increasing stand age. A slight increase at year 6 (0.11) mirrored the transient loss of fine particles during early restoration. The long-term decline in the K factor was driven by the coupling of multiple factors, among which soil organic carbon (OC) played a foundational and pivotal role. The Random Forest model identified OC as the most important predictor variable for K factor variability. This strong association arises because OC acts as an organic binding agent that substantially promotes the formation and stability of soil aggregates, thereby consolidating a stable soil structure and facilitating the retention and sequestration of fine particles. Essentially, enhanced aggregate stability represents the core physical mechanism through which OC reduces soil erodibility [22].

Both principal component analysis and Random Forest analysis revealed that nutrient accumulation and particle-size distribution were strongly related to the dynamics of the soil erodibility K factor. Consistent with this, Ayas [49] recently demonstrated in agricultural ecosystems that improved management practices—such as integrated water, fertilizer, and cover management—can markedly increase soil organic matter content and enhance soil quality. Although conducted in an agricultural setting, this work provides additional support for the broader principle that improving soil quality represents a fundamental mechanism for strengthening ecosystem functions.

The observed relationship between the information dimension (*D*_1_) and the soil erodibility K factor highlights the critical role of particle-size uniformity in erosion resistance. A more homogeneous particle-size distribution limits the pool of readily erodible particles and enhances interparticle packing and cohesion, thereby reducing erodibility. Vegetation characteristics—plant height, cover, and aboveground biomass—exert an indirect ameliorating influence on erodibility by mediating the interception of wind-blown sediments and the input of organic matter.

### 4.4. Study Limitations and Future Applications

This study employed a space-for-time substitution to examine the effects of *S. psammophila* restoration on soil properties. Although widely applied in arid-region ecological research, this approach carries inherent limitations. While it is difficult to guarantee that initial environmental conditions were entirely identical across stands of different ages before restoration, we carefully selected plots to maximize background homogeneity and thereby strengthen the scientific rigor of the study. In addition, our assessment was restricted to the surface soil layer (0–20 cm). In arid and semi-arid regions, deeper soil layers can be influenced by nutrient redistribution driven by deep root exudates, belowground biomass turnover, and precipitation infiltration over prolonged restoration; their response patterns may differ substantially from those observed at the surface. We therefore recommend that future studies incorporate monitoring of deeper soil horizons. In light of the projected intensification of climate-driven aridification and extreme precipitation events, our findings suggest that large-scale vegetation restoration efforts should prioritize protection during the early stages (1–6 years), when the surface is most vulnerable and the risk of erosion is highest.

## 5. Conclusions

In arid and semi-arid dryland ecosystems, vegetation restoration plays a critical role in improving sandy soil texture. The establishment of *Salix psammophila* plantations promotes nutrient enrichment and the deposition of fine particles, ultimately reshaping soil particle-size distribution (PSD) and enhancing erosion resistance. Our results demonstrated that, relative to the control (CK), soil nutrients and fine particle fractions under *S. psammophila* stands improved consistently with increasing stand age, accompanied by progressive soil fining and a sustained decline in pH and sand content. By moving beyond conventional single-index grain-size metrics and applying single-scale and multifractal theory, this study provides a mechanistic understanding of the structural reorganization of soil PSD. The multifractal parameters not only quantified the breadth of the particle-size distribution but also sensitively captured dynamic changes in the uniformity and local heterogeneity of soil particles, confirming the unique diagnostic value of multifractal analysis for assessing soil physical recovery in drylands. With respect to soil erodibility, the random forest model identified soil organic carbon (OC), vegetation growth characteristics, and multifractal parameters (e.g., *D*_1_) as the important predictors of erosion resistance. In management practice, priority should be given to controlling surface wind erosion during the early restoration stage (<6 years). These findings provide new insights into how long-term *S. psammophila* restoration enhances soil structural stability and erosion resistance, and they offer a scientific basis for optimizing vegetation restoration and sustainable desert ecosystem management in arid regions. However, given the inherent limitations of the space-for-time substitution approach, future studies should combine long-term in situ monitoring with deep soil profile analysis to elucidate the ultimate ecological thresholds of *S. psammophila* restoration and the long-term dynamics of soil erodibility.

## Figures and Tables

**Table 1:** Basic information about the research area.

Year	Plant Height (m)	Coverage (%)	Aboveground Herb Biomass (g m^−2^)	Plant Density (Plant m^−2^)	Vegetation Restoration Situation	Organic Carbon (g kg^−1^)	Total Nitrogen (g kg^−1^)	Total Phosphorus (g kg^−1^)	Available Phosphorus (mg kg^−1^)	Alkaline Hydrolysis Nitrogen (mg kg^−1^)	pH
CK	0.00 ± 0.00F	3.50 ± 0.28F	0.18 ± 0.06E	0.00 ± 0.00E	*Agriophyllum squarrosum*	0.47 ± 0.07E	0.04 ± 0.00C	0.16 ± 0.01D	0.96 ± 0.06E	7.92 ± 1.53F	8.71 ± 0.04A
6a	1.70 ± 0.05E	12.99 ± 1.24E	13.39 ± 0.21D	0.05 ± 0.00A	*A. squarrosum*, *Corispermum hyssopifolium*, *Psammochloa villosa*	0.75 ± 0.12DE	0.04 ± 0.00C	0.18 ± 0.02CD	2.87 ± 0.31D	14.12 ± 0.54E	8.75 ± 0.04A
12a	2.33 ± 0.11D	29.58 ± 1.04D	26.14 ± 3.01C	0.05 ± 0.00B	*A. squarrosum*, *C. hyssopifolium*, *P. villosa*, *Grubovia dasyphylla*	0.95 ± 0.27CD	0.05 ± 0.01C	0.20 ± 0.02C	4.18 ± 1.26C	25.45 ± 1.99B	8.57 ± 0.10B
15a	2.58 ± 0.06C	39.40 ± 0.64C	25.67 ± 0.74C	0.05 ± 0.00C	*Artemisia desertorum*, *C. hyssopifolium*, *G. dasyphylla*	1.11 ± 0.38C	0.05 ± 0.01C	0.21 ± 0.06C	2.64 ± 0.34D	19.17 ± 0.38D	8.54 ± 0.15B
25a	2.83 ± 0.060B	45.72 ± 0.90B	33.27 ± 1.21B	0.04 ± 0.00D	*A. desertorum*, *A. squarrosum*, *C. hyssopifolium*, *P. villosa*	1.50 ± 0.10B	0.15 ± 0.00B	0.32 ± 0.03B	5.27 ± 0.69B	21.68 ± 3.97C	8.39 ± 0.02C
35a	2.89 ± 0.02A	56.00 ± 0.52A	40.21 ± 0.86A	0.04 ± 0.00D	*A. desertorum*, *A. squarrosum*, *C. hyssopifolium*, *P. villosa*, *Sophora alopecuroides*	2.28 ± 0.59A	0.20 ± 0.02A	0.38 ± 0.06A	6.05 ± 0.93A	28.76 ± 0.57A	8.19 ± 0.06D

Note: CK denotes mobile dunes with no *S. psammophila* individuals, and plant density was recorded as 0; the herbaceous species listed under vegetation restoration status are those occurring within the plot. Data are presented as mean ± SD (*n* = 10). Different uppercase letters within each column indicate significant differences among stand ages according to Duncan’s multiple range test (*p* < 0.05).

**Table 2:** Soil physical properties under *S. psammophila* plantations of different stand ages.

Year	Soil Water Content (%)	Soil Bulk Density (g cm^−3^)	Clay (%)	Silt (%)	Sand (%)
CK	0.03 ± 0.00A	1.56 ± 0.03A	0.30 ± 0.07C	0.94 ± 0.03CD	98.74 ± 0.10AB
6a	0.03 ± 0.00B	1.55 ± 0.02AB	0.09 ± 0.01D	0.67 ± 0.05D	99.23 ± 0.05A
12a	0.03 ± 0.00AB	1.56 ± 0.02A	0.30 ± 0.13C	1.43 ± 0.34C	98.23 ± 0.46B
15a	0.02 ± 0.00C	1.54 ± 0.01B	0.26 ± 0.10C	2.45 ± 0.61B	97.28 ± 0.70C
25a	0.02 ± 0.00C	1.51 ± 0.03C	0.40 ± 0.09B	2.64 ± 1.32B	96.96 ± 1.37C
35a	0.02 ± 0.01C	1.49 ± 0.03D	1.43 ± 0.18A	6.89 ± 0.42A	91.67 ± 0.24D

Note: Data are presented as mean ± SD (*n* = 10). Different uppercase letters within each column indicate significant differences among stand ages according to Duncan’s multiple range test (*p* < 0.05).

**Table 3:** Distribution characteristics of soil fractal parameters under *S. psammophila* plantations of different stand ages.

Year	D_0_	D_1_	D_2_	D_1_/D_0_
CK	0.87 ± 0.01B	0.62 ± 0.00D	0.50 ± 0.00E	0.71 ± 0.01B
6a	0.82 ± 0.01C	0.64 ± 0.00C	0.54 ± 0.00C	0.71 ± 0.01A
12a	0.85 ± 0.03C	0.59 ± 0.02E	0.51 ± 0.01E	0.70 ± 0.04A
15a	0.86 ± 0.03B	0.64 ± 0.02C	0.53 ± 0.01D	0.75 ± 0.02A
25a	0.91 ± 0.02A	0.71 ± 0.02A	0.58 ± 0.01A	0.78 ± 0.02C
35a	0.91 ± 0.01A	0.69 ± 0.02B	0.56 ± 0.02B	0.75 ± 0.02C

Note: Data are presented as mean ± SD (*n* = 10). Different uppercase letters within each column indicate significant differences among stand ages according to Duncan’s multiple range test (*p* < 0.05).

## Data Availability

The datasets generated during and/or analyzed during the current study are available from the corresponding author on reasonable request.
